# Supplementary material for: The interactome of intact mitochondria by cross-linking mass spectrometry provides evidence for coexisting respiratory supercomplexes
Source: Mol Cell Proteomics. 2017 Dec 8;17(2):216–32. doi: 10.1074/mcp.RA117.000470 (PMC5795388; doi:10.1074/mcp.RA117.000470)
Supplement: Supplemental Data [file supp_RA117.000470_133922_0_supp_23978_fzffwf.zip › spectra_annotation/mito_spectra_annotation/281-2-8-1-8-1.pdf]

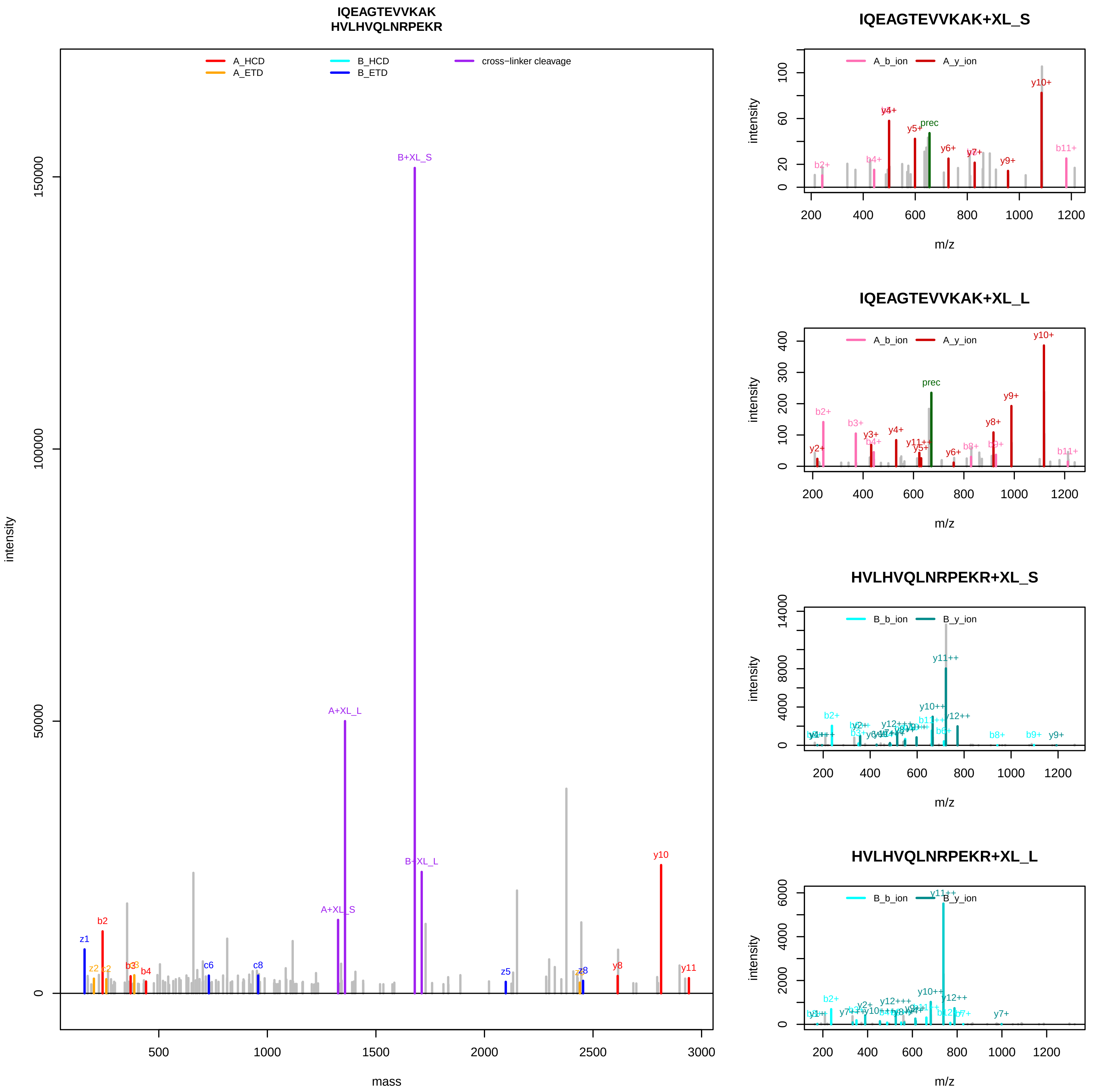

IQEAGTEVVKAK
HVLHVQLNRPEKR
A_HCD
A_ETD
B_HCD
B_ETD
cross−linker cleavage
B+XL_S
A+XL_L
B+XL_L
A+XL_S
y10
y11
y8
b2
b3
b4
z1
z2
c2
c6
c8
z5
z8
intensity
mass
0
50000
100000
150000
500
1000
1500
2000
2500
3000
IQEAGTEVVKAK+XL_S
IQEAGTEVVKAK+XL_L
HVLHVQLNRPEKR+XL_S
HVLHVQLNRPEKR+XL_L
A_b_ion
A_y_ion
B_b_ion
B_y_ion
prec
y10+
y9+
y8+
y6+
y5+
y4+
y3+
y2+
b2+
b3+
b4+
b8+
b9+
b11+
y11++
y10++
y12++
y9+
b9+
b8+
b6+
b2+
y7+
m/z
